# An update on the diversity, ecology and biogeography of the *Saccharomyces* genus

**DOI:** 10.1093/femsyr/foaa013

**Published:** 2020-03-20

**Authors:** Haya Alsammar, Daniela Delneri

**Affiliations:** 1 Department of Biological Sciences, Faculty of Science, Kuwait University, P. O. Box 5969, Safat 13060, Kuwait; 2 Manchester Institute of Biotechnology, Faculty of Biology Medicine and Health, The University of Manchester, Manchester, M1 7DN, UK

**Keywords:** *Saccharomyes* genus, yeast ecology, yeast hybrids, biodiversity

## Abstract

*Saccharomyces cerevisiae* is the most extensively studied yeast and, over the last century, provided insights on the physiology, genetics, cellular biology and molecular mechanisms of eukaryotes. More recently, the increase in the discovery of wild strains, species and hybrids of the genus *Saccharomyces* has shifted the attention towards studies on genome evolution, ecology and biogeography, with the yeast becoming a model system for population genomic studies. The genus currently comprises eight species, some of clear industrial importance, while others are confined to natural environments, such as wild forests devoid from human domestication activities. To date, numerous studies showed that some *Saccharomyces* species form genetically diverged populations that are structured by geography, ecology or domestication activity and that the yeast species can also hybridize readily both in natural and domesticated environments. Much emphasis is now placed on the evolutionary process that drives phenotypic diversity between species, hybrids and populations to allow adaptation to different niches. Here, we provide an update of the biodiversity, ecology and population structure of the *Saccharomyces* species, and recapitulate the current knowledge on the natural history of *Saccharomyces* genus.

## Teaser

The increase in the discovery of wild strains, species and hybrids of the genus *Saccharomyces* has shifted the attention towards studies on genome evolution, ecology and biogeography. Here, we provide an update of the biodiversity and population genomics of the *Saccharomyces* species and recapitulate the current knowledge on the natural history of *Saccharomyces* genus.

## FOREWORDS

The genus *Saccharomyces* includes *S. cerevisiae* a well-known organism in industrial baking and fermentation processes as well as in bioenergy and biomedical fields (Mager and Winderickx 2005; Fukuda, Kondo and Tamalampudi 2009; Sicard and Legras 2011). Since the release of the full genome sequence of *S. cerevisiae* in 1996, extensive functional annotations has started making it the most well-known eukaryotic system to date (Goffeau *et al*. 1996). The availability of a reliable reference genome and the development of high-throughput sequencing subsequently facilitated the whole-genome sequencing and the robust annotation of a large number of *Saccharomyces* species (Cliften *et al*. 2003; Kellis *et al*. 2003; Nakao *et al*. 2009; Scannell *et al*. 2011; Liti *et al*. 2013; Hewitt *et al*. 2014; Baker *et al*. 2015; Naseeb *et al*. 2018). With the increase of ecological surveys of *Saccharomyces* species in nature, these species become models for studies on population genomics (Liti *et al*. 2009; Wang *et al*. 2012; Duan *et al*. 2018; Peter *et al*. 2018). Moreover, robust whole genome sequencing, led to large-scale genomic studies of a variety of strains of *Saccharomyces* species, providing insight into their evolution and natural variation (Warringer *et al*. 2011; Bergstrom *et al*. 2014; Gallone *et al*. 2016; Peter *et al*. 2018).

Research on ecological diversity, population genomics and phenotypic variation for industrial application for both wild and domesticated *Saccharomyces* species have been excelling throughout the last decade. However, the biodiversity and true niche and abundance of the different species remain ambiguous. In this review, we present an overview on the genus *Saccharomyces* focusing on the species biodiversity, ecological niches and population genomics.

## SPECIES OF THE GENUS *SACCHAROMYCES*

**Figure 1.**
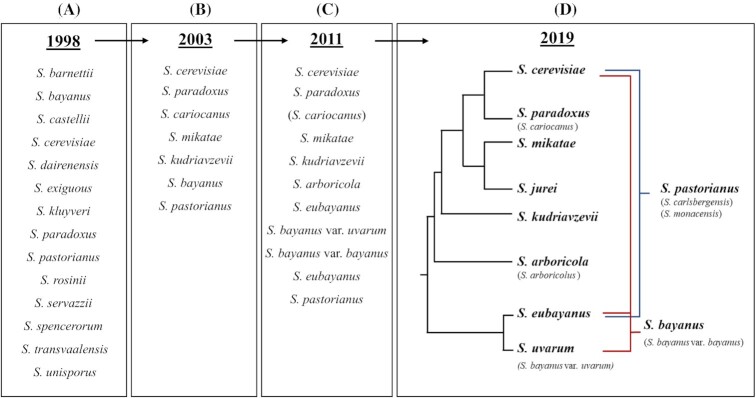
The genus *Saccharomyces* taxonomic rearrangements. The panels show the main changes in the *Saccharomyces* species taxonomy within the *sensu stricto* group over the years. **A)** In 1998, 14 species were included in the ‘sensu stricto’ group (Vaughan-Martini and Martini 1998). **B)** In 2003, several species were reclassified and removed abolishing the group names ‘sensu stricto’ and ‘sensu lato’ (Kurtzman and Robnett 2003). Wild species previously isolated were confirmed as distinct *Saccharomyces* species using molecular and genetic hybridization methods, adding *S. mikatae, S. kudriavzevii* and *S. cariocanus* to the group (Naumov *et al*. 2000b). **C)** From the year 2003 to 2011 further novel species were discovered from nature and other species were reclassified (Naumov 2000a; Wang and Bai 2008; Libkind *et al*. 2011). **D)** Now, the *Saccharomyces* genus consists of eight species and two natural hybrids (Boynton and Greig 2014; Naseeb *et al*. 2017). Previous taxonomical names of the species are in parenthesis.

The name *Saccharomyces* was proposed by J. Meyen in 1838, with *S. cerevisiae* being the first described species. In 1870, M. Reess presented a description of the genus and species that included the yeasts associated with fermentation (Rainieri, Zambonelli and Kaneko 2003). The *Saccharomyces sensu stricto* group was initially described in 1970 in the second edition of ‘The Yeast: A Taxonomic Study’, (Lodder 1970) and originally comprised of 21 species (Teresa Fernandez-Espinar, Barrio and Querol 2003). Over the years, the *Saccharomyces* genus has evolved through taxonomic rearrangements, in which several taxa have been removed and placed in the sister group *Saccharomyces sensu lato* (Fig. 1). In the past, conventional taxonomic methods were employed which had limitations such as the differentiation of strains within a species based solely on morphological and a few physiological characteristics. These limitations have encouraged the integration of molecular methods, such as DNA re-association, chromosomal karyotyping, restriction fragment length polymorphism (RFLP) and sequencing of multiple loci for the classification of *Saccharomyces* species (Vaughan-Martini, Martini and Cardinali 1993; Guillamon *et al*. 1994; Naumov *et al*. 2000b; Kurtzman and Piškur 2006). In 2003, Kurtzman and Barnette established *Saccharomyces* complex as a monophyletic group phylogenetically distinct from *Saccharomyces sensu lato* species. Species of the ‘sensu lato’ group were then reclassified into new species, thus resulting in the termination of the phrases ‘sensu stricto’ and ‘sensu lato’ (Fig. 1A and B, Kurtzman and Robnett 2003). *Saccharomyces* species propagate asexually via budding but are also capable of mating followed by meiosis when the nutrients in the environment become scarce. The presence of sexual reproduction in the yeasts enabled taxonomists to differentiate between the species using the biological species concept (BSC), where only hybridization events within the same species will produce fertile hybrids. Therefore, the production of sterile offspring indicates that the parents belong to two different species (Naumov 1996). The use of BSC has been the method of choice for the taxonomy of budding yeasts given in support of molecular methods. Currently, the advances in DNA sequencing technology allowed quick acquisition of a large amount of genomic data for several species. This enabled a solid resolution of the yeast taxonomy to the strain level and prompted another revision of the classification of the *Saccharomyces* species based on phylogenetic analysis (Fig. 1C).

The genus *Saccharomyces* is now consisting of eight species, namely; *S. cerevisiae, S. paradoxus, S. mikatae, S. jurei, S. kudriavzevii, S. arboricola, S. eubayanus* and *S. uvarum*. Some of these species are parents of natural hybrids that either formed spontaneously in the wild without the involvement of humans or in habitats created by humans e.g. industrial environments (Fig. 1D). All the initially described *Saccharomyces* species were linked to domestication and *S. paradoxus*, the closest relative to *S. cerevisiae*, was the first wild *Saccharomyces* species to be isolated from oak and birch sap in Russia and Ukraine. Based on DNA re-association and genetic hybridization analyses, species that were previously described as *S. cerevisiae* var. *tetrasporus* and *S. cerevisiae* var. *terrestris* are now known as synonyms of *S. paradoxus* (Martini 1989; Naumov 1996). Subsequently, two *Saccharomyces* species were isolated from decayed leaves and soil in Japan and one from the *Drosophila* species in Brazil that were reproductively isolated, with distinct chromosomal profiles, (Naumov, Naumova and Louis 1995a; Naumov *et al*. 1995b). The species isolated from Brazil was described as *S. cariocanus* (now reclassified as *S. paradoxus* based on the low sequence divergence between the species), while the two species from Japan were described as *S. kudriavzevii* and *S. mikatae* (Naumov *et al*. 2000b). In 2008, *S. arboricola* was isolated from the bark of broadleaf trees in China (Wang and Bai 2008).

The classification of *S. uvarum* and *S. bayanus* was controversial and went through several revisions (Naumov 1996; Nguyen and Gaillardin 1997; Nguyen, Lepingle and Gaillardin 2000). *S. bayanus* has been recognized as a complex cryotolerant species separated into two varieties; the heterogenous strains belonging to *S. bayanus* var. *bayanus* and the homogenous strains *S. bayanus* var. *uvarum* (Vaughan-Martini and Martini 2011). *S. bayanus* var. *uvarum* consist of a pure lineage strain with no genomic contribution from other *Saccharomyces* species, thus is now known as a distinct species named *S. uvarum* (Fig. 1C, Rainieri *et al*. 1999; Nguyen, Lepingle and Gaillardin 2000; Pulvirenti *et al*. 2000). Recently, the separation of *S. bayanus* into two varieties based on the BSC is considered taxonomically invalid and *S. uvarum* stands as s real species and not a variety of *S. bayanus* (Nguyen and Boekhout 2017). However, *S. bayanus* var. *bayanus* is now recognized as a natural hybrid rather than a true species. The isolation of *S. eubayanus* from a southern beech (*Nothofagus* spp.) tree in Patagonia, Argentina resolved the taxonomic classification of *S. bayanus*. A comparative genomic analysis revealed that the *S. bayanus* CBS 380^T^ genome is composed of 67% *S. uvarum* and 33% *S. eubayanus* sequences with introgressions from *S. cerevisiae*, making *S. bayanus* a hybrid between these three species (Fig. 1D, Libkind *et al*. 2011). The latest addition to the *Saccharomyces* genus is *S. jurei*, which was isolated from oak bark and the surrounding soil in the pre-Alps near Saint-Aubin, France. This species is reproductively isolated and phylogenetically distinct from all members of the *Saccharomyces* species. *S. jurei* is genealogically closely related to *S. mikatae, S. paradoxus* and *S. cerevisiae* based on sequences of the internal transcribed region (ITS1–5.8S-ITS2) and the D1/D2 domains of the 26S rRNA (Naseeb *et al*. 2017). Whole-genome sequencing and phylogenetic analyses of a concatenation of 101 universally distributed orthologs placed *S. jurei* and *S. mikatae* in a monophyletic group. In addition, the *S. jurei* species possesses two chromosomal translocations, one of which is shared with the two *S. mikatae* strains IFO1815 and IFO1816, suggesting a common evolutionary history (Naseeb *et al*. 2018).

## BIOGEOGRAPHY OF *SACCHAROMYCES* SPECIES

### Saccharomyces cerevisiae

The phylogenetic analysis of wild and domesticated *S. cerevisiae* strains has revealed a complex population structure (Fay and Benavides 2005; Liti *et al*. 2009). The first population genomic studies used genome wide single nucleotide polymorphisms (SNPs) analysis to cluster *S. cerevisiae* strains into five delineated populations that correlated with isolation regions and fermentation types: North American, Malaysian, West African, sake and wine/European (Table 1). However, some strains (primarily human-related) were not assigned to a specific lineage and were labelled as mosaic due to the polymorphic nature of their genome (Liti *et al*. 2009). A further study surveying New Zealand habitats found seven distinct *S. cerevisiae* subpopulations isolated from soil, bark, flowers and spontaneous ferment (Goddard *et al*. 2010). Interestingly, the New Zealand strains are phylogenetically closely related to the European population, as shown by the number of shared alleles (Cromie *et al*. 2013). Another large-scale field survey of primeval forests in China resulted in the isolation of 99 wild *S. cerevisiae* strains belonging to eight distinct lineages that were partially reproductively isolated (10.2% to 89.1% spore viability) (Wang *et al*. 2012). More recently, genome-wide SNPs analyses of over 200 wild and domesticated Chinese strains revealed two new wild lineages increasing the number of the Chinese populations to twelve (Duan *et al*. 2018). Phylogenetic analysis of the Chinese strains and *S. cerevisiae* of worldwide origins revealed one of the Chinese populations to be the most ancient, forming the basal lineage of the phylogenetic tree. The high number of genetically diverged lineages present in this region indicated that the species had an Asian origin (Duan *et al*. 2018). Such view has been recently supported by Peter and co-workers in their analysis of SNPs in 1011 *S. cerevisiae* strains of domesticated, human and wild origins using statistical dimension reduction tools, which supported the hypothesis of an origin of this species outside China (Peter *et al*. 2018).

**Table 1.** Common niches and global distribution of the wild *Saccharomyces* populations.

Species	Ecology	Populations
*S. cerevisiae*	Broadly associated with bark and soil Fagales order trees	Asian, European, North American and South American
*S. paradoxus*	Broadly associated with bark and soil of *Qurecus* spp.	Asian, European, North American (America A/Europe, America B and America C)
*S. eubayanus*	Broadly associated with *Nothofagus* spp.	Patagonian A, Patagonian B/Holarctic (North America and Tibet strains) and West Chinese
*S. uvarum*	Broadly associated with *Nothofagus* spp. and other Fagales spp.	South American A/Holarctic, South American B and Australasian
*S. kudriavzevii*	Decayed leaf, soil, bark of mainly *Quercus* spp.	European (Portugal, Spain & France) and Asian (Taiwan and Japan)
*S. arboricola*	The bark of *Quercus fabri, Castanopsis orthacantha*, soil and seeds, Fruiting body of *Auricularia polytricha*	Asian (China and Taiwan) and Australasian (New Zealand)
*S. mikatae*	Soil and decayed leaf	Asian (Japan)
*S. jurei*	Bark and soil of *Quercus robur*	European (France)

A distinct monophyletic lineage of a wild population of *S. cerevisiae* associated with Mediterranean oak (MO) was only detected in southern Europe (Almeida *et al*. 2015). The MO population is closely related to the wine population based on genome wide analysis. Strains of the wild MO population were shown to be the source of the ancestor domesticated strains (wine strains) based on population demographics analysis (Almeida *et al*. 2015). However, Duan *et al*. (2018) proposed that the wine strains originated in Asia as proven by clustering a few wild Chinese isolates with the wine lineage and sharing horizontally transferred genes between strains of the two populations.

A novel South American population was isolated from Brazil and grouped into a single clade that is clearly separated from the other previously known populations. Some of these strains displayed a mosaic genome, and 54% of the Brazilian strains had only a small amount of introgression from the wine population strains, suggesting a previous domestication in the history of *S. cerevisiae* (Barbosa *et al*. 2016). More sampling in a systematic way which will encourage the exploration of undescribed *Saccharomyces* populations.

### Saccharomyces paradoxus

In contrast to *S. cerevisiae, S. paradoxus* has been almost completely limited to wild environments, and forms well-structured populations that are related to a geographic origin and that are less phenotypically diverse than *S. cerevisiae* (Liti *et al*. 2009, Warringer *et al*. 2011). *S. paradoxus* strains were originally designated into three geographically-structured populations: Far Eastern, European and North American, with a less defined Hawaiian population represented by a single strain (Liti, Barton and Louis 2006; Liti, Barton and Louis 2009) (Table 1). These populations are partially reproductively isolated and are diverged by 1.5% to 4.6% (Liti, Barton and Louis 2006). The North American population is further divided into three lineages: America A/Europe, America B and America C, (Table 1). The America A/Europe lineage includes European strains that are thought to have recently migrated to North America. The American populations shows about 2.0% to 3% inter-lineage nucleotide divergence based on the genes *POP2* and *RPB2* (Leducq *et al*. 2014). These lineages co-exist in partial sympatry in North America, showing secondary contact of original populations that diverged allopatrically (Kuehne *et al*. 2007; Leducq *et al*. 2014). The secondary introduction of a diverged population also expanded the geographical distribution of the European population. *S. paradoxus* strains that are highly similar to the European ones have also been detected in New Zealand; it has been proposed that the European strains were introduced to the region through the shipment of oak acorns from Australia or the United Kingdom (Zhang *et al*. 2010). In addition to the cases of occupancy overlap with the America A and B lineages, the American lineages are generally broadly separated along a north-south gradient in North America. The lineages show phenotypic divergence reflecting the differences in their ability to adapt to local temperature that influenced their distribution (Leducq *et al*. 2014). Partial post-zygotic isolation has been demonstrated within and between the genetically and phenotypically diverged North American populations that were associated with chromosomal rearrangements, indicating the early stages of speciation (Charron, Leducq and Landry 2014a).

### Saccharomyces eubayanus

*S. eubayanus* strains were initially isolated in Patagonia (Argentina) and are clustered into two lineages: Patagonia A and Patagonia B. A few strains that were later isolated from North America (Wisconsin) were identified as being a mixture of the two lineages (Table 1). The Patagonia B lineage is diverged from the Patagonia A lineage, revealing a divergence of 0.93%, based on the sequences of nine nuclear genes and a mitochondrial gene (Peris *et al*. 2014). A single *S. eubayanus* strain that was isolated from New Zealand was clustered with the Patagonia B lineage, according to the phylogenetic analyses of six loci (Gayevskiy and Goddard 2016). The distribution of *S. eubayanus* has extended to Far East Asia, where three lineages have been discovered in different regions of China: West China, Sichuan and Tibet/Lager. The genetic diversity within the Asian population is up to 7.57% (multilocus analysis may overestimate sequence divergence between species in comparison to genome-wide analysis), which was higher than what has been recorded between the Patagonia A and B lineages (Bing *et al*. 2014). Multilocus phylogenetic analyses of previously known strains and of strains from North America (Washington, North Carolina and Canada) have identified a new clade that includes strains with a Holarctic distribution genetically closely related to the Patagonia B population (0.56% genetic distance based on the complete genome). Based on the latest molecular analyses, the three main *S. eubayanus* populations have been recognized as Patagonia A, Patagonia B/Holarctic including strains from North America and Tibet and West Chinese (Peris *et al*. 2016a). Extensive sampling of *Nothofagus* sp. trees in South America revealed a uniquely high isolation frequency of *S. eubayanus* strains and genome-wide sequencing added depth to the phylogeny of the specie populations (Eizaguirre *et al*. 2018; Langdon *et al*. 2019; Nespolo *et al*. 2019). Adding to the complexity of the *S. eubayanus* populations, six sub-populations are now recognized (PA1, PA2, PB1, PB2, PB3 and Holarctic) in addition to admixture populations (Langdon *et al*. 2019).

### Saccharomyces uvarum

The whole-genome data of the *S. uvarum* strains that are associated with wild and domesticated environments in North and South America, Eurasia and Australasia have been phylogenetically analysed and grouped into three clades: South American A/Holarctic, South America B and Australasia (Table 1) (Almeida *et al*. 2014). The South American A/Holarctic clade primarily includes strains that have been isolated from Holarctic regions, along with a few South American strains, while the B clade only contains South American strains. The Australasian lineage is distinctly separated from the other populations, with 4.4% genome divergence, and is partially reproductively isolated from the other *S. uvarum* strains. The highest level of species diversity has been found in the Southern Hemisphere, where two populations have diverged by 1%. This high level of diversity was demonstrated by the pairwise nucleotide diversity of the South American isolates compared to the Holarctic and Australasian isolates (0.689 vs 0.141 and 0.162, respectively). The low diversity of the Holarctic isolates and the phylogenetic grouping of the strains within the South American A lineage suggests that the Holarctic population is derived from the South American A population and only recently migrated into the Northern Hemisphere (Almeida *et al*. 2014).

### Saccharomyces kudriavzevii

The *S. kudriavzevii* species is currently represented by Asian strains that have been isolated from Japan and Taiwan and European strains that have been isolated from Portugal , Spain and France (Table 1) (Naumov *et al*. 2000b; Sampaio and Goncalves 2008; Lopes, Barrio and Querol 2010; Erny *et al*. 2012; Naumov, Lee and Naumova 2013). Multilocus sequence analyses of the *S. kudriavzevii* strains that have been isolated from Europe (Spain and Portugal) have revealed that the strains are closely related, with a nucleotide diversity of 0.21%. These strains are diverged by 0.51% from the Japanese type strain (IFO 1802^T^); consequently, they were assigned to an Iberian/European population (Peris *et al*. 2016b). Based on genome-wide sequencing analysis, a single Japanese *S. kudriavzevii* strain (IFO 1803) was shown to be diverged from the other known strains by ∼4% (Hittinger *et al*. 2010). Recently, a large number of *S. kudriavzevii* strains were isolated from the Italian Carnic Alps that showed phenotypic variation (Alsammar 2018). These strains are closely related to the European strains (CA111 and ZP629) based on multiloci analysis, but form a distinct sub-population based on whole genome SNPs analysis (Alsammar 2018). A feature that distinctly differentiates the European strains from the Asian strains is the ability to utilize galactose of the former. The Japanese strains have retained pseudogenes of the seven *GAL* pathway genes, but they are heavily mutated, rendering them non-functional (Hittinger, Rokas and Carroll 2004; Hittinger *et al*. 2010). The previous population genomics study of *S. kudriavzevii* did not include the Taiwanese strains, however, phylogenetic analyses of the D1/D2 and ITS1 sequences clustered most of the Taiwanese strains with the Japanese IFO 1803 strain, while others were grouped with the Portuguese strains and the Japanese type strain IFO 1802^T^ (Naumov, Lee and Naumova 2013). Interestingly, the distribution of *S. kudriavzevii* seems to be restricted to Europe and Asia, since it has not been isolated from other regions that are densely populated with well-structured populations of *Saccharomyces* species, such as North or South America. A comprehensive population genomics study for this species that includes all the strains that have been isolated from the different regions has not yet been conducted, however it seems clear that *S. kudriavzevii* strains are grouped into an Asian population (that includes the Japanese and Taiwanese strains) and a European population composed of the strains that have been isolated from Portugal, Spain and France (Table 1).

### Saccharomyces arboricola

To date, the distribution of *S. arboricola* has been limited to Far East Asia (China and Taiwan) and Australasia (New Zealand, Table 1) (Wang and Bai 2008; Naumov, Lee and Naumova 2013; Gayevskiy and Goddard 2016). The Chinese strains closely resemble the Taiwanese strains, as the type strain exhibits ITS and D1/D2 sequences that are identical to the Taiwanese strains (Wang and Bai 2008; Naumov, Lee and Naumova 2013). Nine *S. arboricola* strains that were isolated from soil in New Zealand possess a genome divergence of 2.6% from a Chinese reference strain (Gayevskiy and Goddard 2016).

### *Saccharomyces mikatae* and *Saccharomyces jurei*

*S. mikatae* has only been isolated in Japan, and it encompasses two strains, IFO 1815^T^ and IFO 1816 (Table 1) (Naumov *et al*. 2000b). Similarly, *S. jurei* has been found only in Europe, with two strains, NCYC 3947^T^ and NCYC 3962, isolated from oak bark and soil, respectively, in the French pre-Alps (Naseeb *et al*. 2017).

## *SACCHAROMYCES* INTERSPECIFIC HYBRIDS

**Figure 2.**
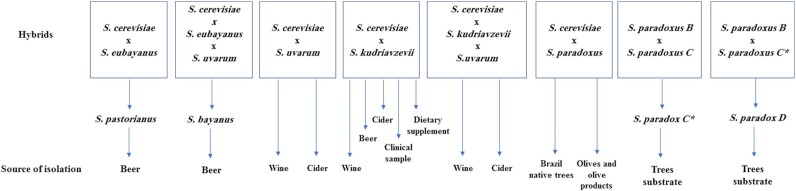
Common *Saccharomyces* hybrids and the source of their isolation. *Saccharomyces* may hybridize forming double or triple hybrids that are of industrial significance. Most of the known hybrids are associated with domestication activities and a few strains isolated from non-fermentation environments.

Species of *Saccharomyces* readily hybridize due to the absence of significant prezygotic barriers, and produce hybrids that are sterile primarily due to sequence divergence among the species (Morales and Dujon 2012). Hybrids among *Saccharomyces* species are common in industrial fermentation environments involved in brewing and wine making process (Fig. 2) (Sicard and Legras 2011), however, they are scarcely reported in wild (Barbosa *et al*. 2016) and medical samples (Peris *et al*. 2018). Hybridization is advantageous in *Saccharomyces* evolution, since it introduces high genetic variation leading to novel lineage conferring hybrid vigour and wider adaptation potential (Gonzalez *et al*. 2007; Belloch *et al*. 2008; Piatkowska *et al*. 2013).

The most well-known industrial hybrid is *S. pastorianus*, resulting from the cross between *S. cerevisiae* and *S. eubayanus* (Fig. 1D, syn. *S. carlsbergensis*). This hybrid has been used for centuries in brewing and is responsible for lager production, which is conducted at low temperatures (5–14°C), in contrast to ale brewing which occurs at higher temperatures (15–24°C) and is carried out by *S. cerevisiae* (Sicard and Legras 2011). The cold-tolerant nature of *S. pastorianus* allows the species to ferment at low temperatures, a trait inherited from the *S. eubayanus* parent; meanwhile, the *S. cerevisiae* sub-genome contributes to the hybrid's ability to ferment maltotriose (Hebly *et al*. 2015).

Array comparative genomic hybridization analysis of several *S. pastorianus* strains identified two distinct lineages, based on differences in chromosome content, chromosome structure and ploidy, namely; Saaz-type (group 1) and Frohberg (group 2), named after the region of initial isolation and the region of brewing, respectively (Dunn and Sherlock 2008). The origin of the *S. eubayanus* lager yeast parent was thought to be South American, due to the high abundance of this species in that region, introduced to European brewing after early trans-Atlantic trade (Libkind *et al*. 2011). However, brewing originated in Bavaria during medieval time and rapidly expanded in the 1400s, long before the beginning of the trans-Atlantic trade in the 1500s. Following the *S. eubayanus* discovery in Patagonia, Asian populations of the species were isolated from various regions in China, and the genome of a Tibetan strain was shown to be 99.82% similar to the *S. eubayanus* subgenome of the lager yeast making it the more likely parent of the lager yeasts, with *S. cerevisiae* being the other parent. This discovery led scientists to hypothesize that *S. eubayanus* was introduced to Europe through the silk road (Bing *et al*. 2014). However, genome-wide pairwise nucleotide sequence divergence analysis revealed regions in the Tibetan strains that are more similar to North Carolina strains than to *S. pastorianus*, which was also supported by phylogenetic analysis (Peris *et al*. 2016a). Based on these findings Peris *et al*. (2016a) concluded that none of the known *S. eubayanus* is with certainty the nearest parent of *S. pastorianus.S. eubayanus* has still not been isolated in Europe, although DNA signals of the species were detected in soil of Italian mountain regions (Alsammar *et al*. 2019).

**Figure 3.**
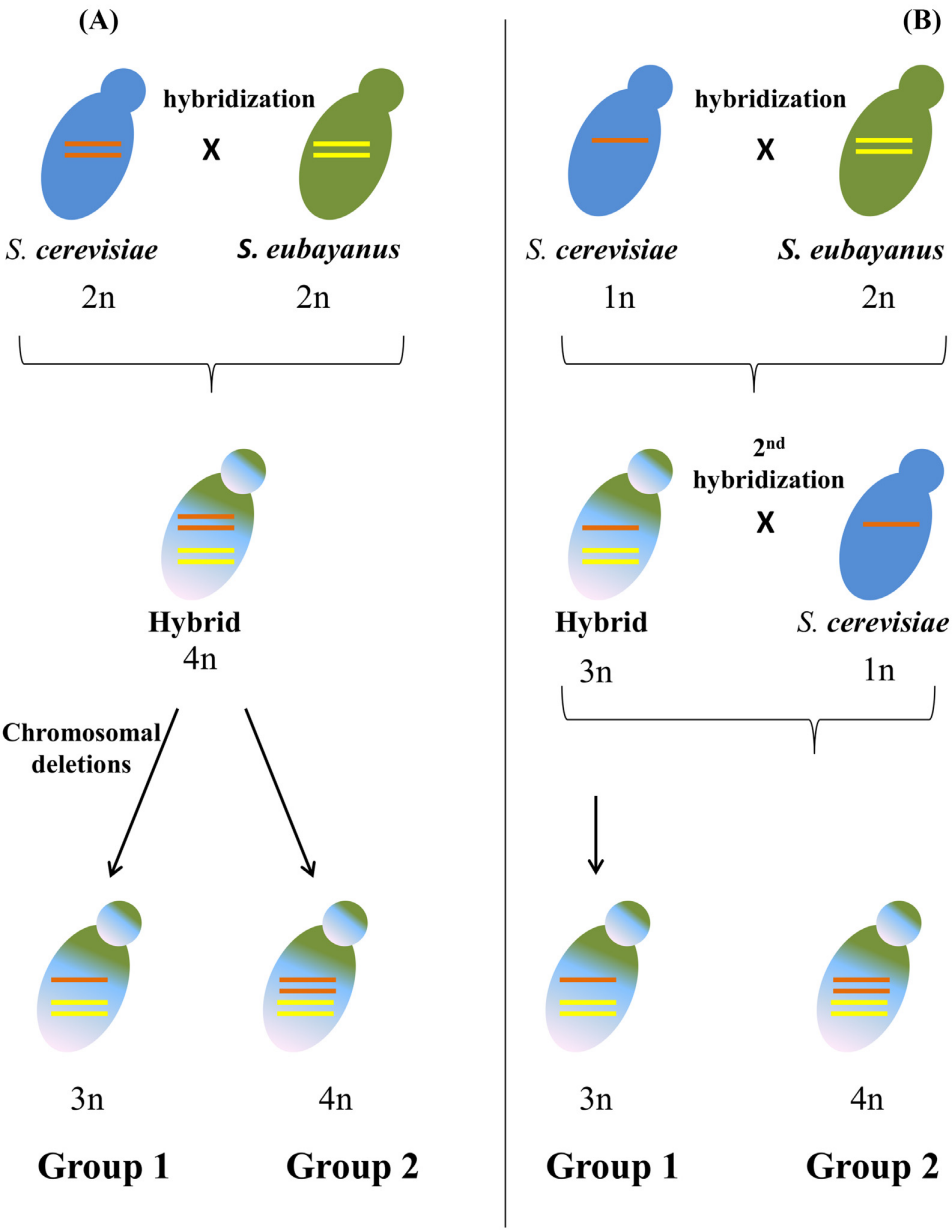
Origin of *S. pastorianus* group 1 and group 2 strains based on two theories. (A) hybridization between diploid *S. cerevisiae* and *S. eubayanus* followed by chromosomal deletions in the *S. cerevisiae* sub- genome of group 1 strains. (B) The hybridization of a haploid *S. cerevisiae* and a diploid *S. eubayanus* lead to a triploid hybrids (3n) followed by a second hybridization event in group 2 strains (4n) (Figure adapted from Okuno *et al*. (2016).

The genetic differences between group 1 and group 2 lager yeasts was explained by independent hybridization of group 1 and group 2 lager hybrids (Monerawela *et al*. 2015). However, the presence of conserved chromosomal translocation events in strains of both groups suggest a common ancestor (Walther, Hesselbart and Wendland 2014; Okuno *et al*. 2016). The latest SNPs analysis by Okuno *et al*. (2016) sheds light on the evolution of the lager yeasts, which suggests at least a single common hybridization event between the groups. The authors proposed two possible theories to explain the hybridization origin of the lager yeasts (Fig. 3): 1. A common ancestor originates from the hybridization of a diploid ale-type *S. cerevisiae* and a diploid *S. eubayanus* resulting in group 2 (4n) strains. Chromosomal deletions in *S. cerevisiae* genome of the 4n hybrid gave rise to to group 1 (3n) strains (Fig. 3A). 2. An initial hybridization of a haploid ale-type *S. cerevisiae* with a diploid *S. eubayanus* producing the ancestral group 1 (3n) yeasts, followed by a second hybridization with haploid *S. cerevisiae* strain resulting in the ancestor of group 2 (4n) yeasts (Fig. 3B).

Genetic analysis of beer, wine and cider *Saccharomyces* strains lead to the discovery of other natural double interspecific hybrids (Fig. 2), *S. cerevisiae* x *S. uvarum* and *S. cerevisiae* x *S. kudriavzevii*, including triple hybrids, *S. cerevisiae* x *S. kudriavzevii* x *S. uvarum* (Masneuf *et al*. 1998; Bradbury *et al*. 2006; Gonzalez *et al*. 2006; Lopandic *et al*. 2007; Gonzalez, Barrio and Querol 2008; Peris *et al*. 2012a). *S. cerevisiae* x *S. kudriavzevii* hybrids were also isolated from clinical samples and dietry supplement (Peris *et al*. 2012a).

Phylogenetic analysis of the European *S. kudriavzevii* strains showed that they are more closely related to the natural *S. cerevisiae* x *S. kudriavzevii* hybrids (associated with fermentation in central Europe) than the Asian strains, thus indicating an hybridization of European origin (Sampaio and Goncalves 2008; Lopes, Barrio and Querol 2010). Unlike *S. cerevisiae, S. kudriavzevii* has not been found in fermentative environments, suggesting that the hybridization event between *S. cerevisiae* and *S. kudriavzevii* may have taken place in the wild before the hybrids expanded to domesticated settings (Belloch *et al*. 2009).

The proportion of *S. kudriavzevii* genome relative to *S. cerevisiae* genome in the hybrids differs between strains (Belloch *et al*. 2009; Erny *et al*. 2012; Peris *et al*. 2012b; Borneman *et al*. 2016). The hybrids with a higher proportion of *S. cerevisiae* sub-genome are better adapted to fermentation stresses, while the hybrids with higher amount of *S. kudriavzevii* sub-genome are more efficient at fermentation at low temperature (Belloch *et al*. 2008; Peris *et al*. 2012b).

Other hybrids have also been isolated in the wild such as those between *S. cerevisiae* x *S. paradoxus* (Barbosa *et al*. 2016). The clear introgressions in *S. cerevisiae* coming from *S. paradoxus* genome support the occurrence of hybridization of these two species in the wild (Barbosa *et al*. 2016). A considerable number of open reading frames (ORFs) belonging to *S. paradoxus* were recorded to be introgressed in the genomes of *S. cerevisiae* analysed by Peter et al 2018. Recently, *S. cerevisiae* x *S. paradoxus* hybrids were isolated from processed olives and olive products (Fig. 2). These hybrids in addition to other *S. cerevisiae* x *S. paradoxus* previously isolated from the similar substrates formed a distinct lineage named the ‘olives clad’(Pontes *et al*. 2019).

Genetic analysis of the North American *S. paradoxus* lineages that are partially sympatric revealed hybridization events within the natural lineages suggesting the occurance of hybridization in nature. The genome of the hybrid species (SpC*) is a mosaic of the North American lineage *SpB* and *SpC* genotypes due to the secondary contact between the parental lineages.The phenotypic growth response of the hybrid lineage is unique, corresponding to conditions of the contact region between the hybrid's parents (Leducq *et al*. 2016). Recently, novel intraspecific hybrids (*SpD*) generated between backcrossing of the hybrid species SpC* and its parental lineage *SpB* were isolated from natural environments. *SpD* hybrids revealed partial reproduction isolation with the North American lineages and a distinct growth and transcriptome profiles, thus leading to the increasing chance of hybrid formation and persistence in nature (Eberlein *et al*. 2019).

## ECOLOGY OF *SACCHAROMYCES* SPECIES

The fermentation processes of domesticated *Saccharomyces* species have been thoroughly studied, leading early ecological studies to investigate fermentation-related environments, such as breweries and vineyards, as the typical habitats of *Saccharomyces* species (Sampaio and Gonçalves 2017). However, most *Saccharomyces* species are now recognized as being wild species that are isolated from environments not related to human activity (Naumov, Naumova and Sniegowski 1998; Naumov 2000a; Wang and Bai 2008; Libkind *et al*. 2011; Naseeb *et al*. 2017). Some species are present in both wild habitats and domesticated environments (Almeida *et al*. 2014; Peter *et al*. 2018). The ecology of *S. cerevisiae* extends to human guts and may be correlated to disorders such as irritable bowel syndrome (Nash *et al*. 2017; Sokol *et al*. 2017). The differentiation between wild and domesticated *Saccharomyces* populations reflects distinct genomic evolutions history shown by differences in chromosomal cores and subtelomeres (Yue *et al*. 2017).

The hybrids *S. pastorianus* and *S. bayanus* have not been isolated from natural environments and are strictly associated with brewing environments (Rainieri *et al*. 2006). Subsequently, they were maintained due to brewing-related selection pressures (Dunn and Sherlock 2008; Libkind *et al*. 2011). *S. paradoxus, S. mikatae, S. jurei, S. kudriavzevii, S. arboricola* and *S. eubayanus* are purely wild species, while *S. cerevisiae* and *S. uvarum* encompass domesticated and wild strains . The wild species are commonly associated with tree substrates, such as bark, soil, leaves, exudates and litter. The frequent isolation of *Saccharomyces* species, especially *S. paradoxus* and *S. cerevisiae*, from *Quercus* spp. (oak) led to the hypothesis that this particular tree is the yeasts’ natural habitat (Naumov, Naumova and Sniegowski 1998; Sniegowski, Dombrowski and Fingerman 2002; Johnson *et al*. 2004; Sampaio and Goncalves 2008; Hyma and Fay 2013; Charron *et al*. 2014b). However, *Saccharomyces* species have also been isolated from several other tree species (Table 1), extending the their habitat to the order Fagales (Sampaio and Goncalves 2008; Libkind *et al*. 2011; Alsammar 2018). In fact, the absence of *Quercus* spp. from South America encouraged the exploration of native tree species, such as *Nothofagus* sp. (Southern beech, a member of the order Fagales), as well as the sugar-rich fruiting stromata of *Cyttaria hariotii* (a tree parasite) which resulted in the isolation of *S. eubayanus* and *S. uvarum* (Libkind *et al*. 2011). These species have also been isolated from *Araucaria araucana*, a native South American tree (Rodriguez *et al*. 2014). The presence of these species in the Southern Hemisphere is correlated with the native tree species, suggesting that the species are well-established in this region (Rodriguez *et al*. 2014). In contrast, *S. uvarum* has been isolated in at low frequency, primarily from *Quercus* spp. in Europe and was also isolated from the *Nothofagus* that are present in New Zealand and Tasmania (Almeida *et al*. 2014). *S. eubayanus* have been detected in North America and China, primarily associated with *Quercus* spp. (Bing *et al*. 2014, Peris *et al*. 2016a), while the isolation of a single *S. eubayanus* in New Zealand was from sampling fruits, bark and soil of trees that were native to the region (Gayevskiy and Goddard 2016).

Oak trees are the most common host for *Saccharomyces* species in the Northern hemisphere. *S. paradoxus* specifically is frequently isolated from oak bark, soil and exudates; in some cases, this species has been isolated in sympatry with *S. cerevisiae* (Naumov, Naumova and Sniegowski 1998; Sniegowski, Dombrowski and Fingerman 2002; Sampaio and Goncalves 2008; Sampaio and Gonçalves 2017). Large ecological surveys of *Saccharomyces* species have demonstrated the specificity of *S. paradoxus* to oak trees. Tha majority of trees sampled from different regions in Canada harboured a 3-fold higher percentage of *S. paradoxus* compared to other tree species (Charron *et al*. 2014b). Sampling of various trees in the United States has also revealed a significant association of *S. paradoxus* with oak trees (Sylvester *et al*. 2015). However, neither of these studies successfully isolated *S. cerevisiae*, whose presence may have been restricted by the northern limit of the sampling regions. Both species differ in their thermal growth profiles, with *S. cerevisiae* having a higher optimum temperature than *S. paradoxus*. Therefore, the absence of *S. cerevisiae* may have been affected by lower temperatures of the sampling areas (Sweeney *et al*. 2004; Salvado *et al*. 2011). A possible explanation for the general frequent isolation of *Saccharomyces* species from trees bark is that the species might seek refuge in the tree bark during seasonal changes (Goddard and Greig 2015). Despite the common association of *Saccharomyces* species with oak tree bark, Kowallik and Greig (2016) showed that samples of leaf litter surrounding oak trees yielded a higher abundance of *S. paradoxus* than from bark suggesting that the yeasts may be dispersed from tree bark to litter by rainwater or insects (Kowallik and Greig 2016). The tree bark niche for the *Saccharomyces* species is not fully understood, as the sugar content of this habitat is too low to support the growth of Crabtree-positive yeast species (Boynton and Greig 2014). The presence of *Saccharomyces* species on bark has been correlated with the presence of hexoses sugars, which may explain the species’ occurrence (Sampaio and Goncalves 2008). Analysis of the human gut microbiome revealed the abundance of *S. cerevisiae* found in 92.2% of the sampled volunteers, indicating that the species is a common resident of the gut (Nash *et al*. 2017). A shift in the abundance of *S. cerevisiae* in the human gut was shown to be associated with inflammatory bowel disease microbiota dysbiosis (Sokol *et al*. 2017).

*S. uvarum* is associated with wine and cider fermentation, however, it is not considered to be fully domesticated, as strains have been isolated from several natural environments (Sampaio and Goncalves 2008; Libkind *et al*. 2011). Although the numbers of *S. uvarum* isolates are generally low in comparison to other species, they have a global distribution, with the Southern Hemisphere harbouring a high abundance of the species (Almeida *et al*. 2014). Similarly, *S. cerevisiae* has a global distribution being isolated from natural environments in North America, China and Europe, as well as domesticated ones such as vineyards, fruits and insects (Sniegowski, Dombrowski and Fingerman 2002; Stefanini *et al*. 2012; Wang *et al*. 2012; Hyma and Fay 2013; Almeida *et al*. 2015). Phylogenetic analysis of *S. cerevisiae* species has revealed that the wild strains have the oldest lineages and are located at the root of the phylogenetic tree; moreover, wild strains have a higher genetic diversity than most domesticated strains, suggesting that the domesticated strains are derived from the natural populations (Fay and Benavides 2005; Wang *et al*. 2012; Almeida *et al*. 2015). The association of *S. cerevisiae* with *Drosophila* spp., bees and wasps, especially in regions that are populated with fruits, represents a source of the yeast's dispersal that maintains genetic diversity and protection during unfavourable seasonal climates (Goddard *et al*. 2010; Stefanini *et al*. 2012; Buser *et al*. 2014).

Despite the enrichment culture's sensitivity for the isolation of the *Saccharomyces* species from environmental samples (Kowallik, Miller and Greig 2015), the method may introduce biases toward the isolation of one or a few species that can outcompete others in the selection media. If the *Saccharomyces* species are outgrown by other species in the sample, the actual species distribution may be underestimated (Boynton and Greig 2014; Goddard and Greig 2015). Moreover, the enrichment culture method will not reveal the actual abundance of the species in a natural environment, as a single cell might propagate, forming cell clones and lead to an overestimation of the species’ existence. For example, Kowallik, Miller and Greig (2015) reported that *S. paradoxus* was rare on oak bark, as demonstrated when the bark samples were inoculated in a malt extract medium that had been supplemented with lactic acid and was outcompeted by surrounding microbial species.

Differences in growth temperatures of the *Saccharomyces* species influence their ecological interactions in nature. Wild species with different temperature growth profiles have been reported to occupy the same habitat (Sweeney *et al*. 2004; Sampaio and Goncalves 2008; Paget, Schwartz and Delner 2014) such as the coexistence of *S. paradoxus* and *S. cerevisiae* on oak bark from a single sampling site in North American (Sniegowski, Dombrowski and Fingerman 2002). Moreover, the incubating tree bark at high (30°C) and low temperatures (10°C) resulted in the isolation of *S. cerevisiae* coupled with *S. kudriavzevii* and *S. paradoxus* and with *S. uvarum* (Sampaio and Goncalves 2008). The thermo-niche adaptation is due to differences in optimal growth temperatures and circadian temperature changes that allows the alternating growth of the species, thus preventing the abundance of one species over the other.

## DNA SIGNALS OF *SACCHAROMYCES* SPECIES IN NATURE

To avoid culturing biases and to determine the actual abundance of the *Saccharomyces* species in their natural habitat a high-throughput sequencing of environmental DNA (eDNA) extracted from bark, soil and vineyard samples was employed by several research groups (Taylor *et al*. 2014; Kowallik, Miller and Greig 2015; Dashko *et al*. 2016; Alsammar *et al*. 2019). Pyrosequencing of bark samples and bark infusions did not result in the detection of any of the *Saccharomyces* species (Kowallik, Miller and Greig 2015). High-throughput sequencing of grapes collected from vineyards of different regions in New Zealand yielded only *S. cerevisiae* at an abundance of 1:20 000 (Taylor *et al*. 2014). DNA signatures of *S. cerevisiae, S. paradoxus, S. mikatae* and *S. pastorianus* were detected in oak bark and soil of vineyard trees and wine must samples in Slovenia. The *Saccharomyces* were rare in the bark and soil samples, however, *S. cerevisiae* and *S. paradoxus* were the dominant species in must samples (Dashko *et al*. 2016). Targeting *Saccharomyces* eDNA based on the size of the ITS region extracted from soil surrounding different tree species at varying altitudes succeeded in the detection of most species of the *Saccharomyces* species in low abundance in comparison to other fungi (Alsammar *et al*. 2019). Although *S. mikatae* was not isolated outside Asia, metagenomic signature of the species has been detected in grape must in Europe (Dashko *et al*. 2016) and in soil surrounding oak, spruce and beech trees (Alsammar *et al*. 2019), suggesting a wider distribution of the species. Also, *S. jurei* has not yet been isolated from areas other than its original isolation region. However, eDNA of this species was detected in soil surrounding different tree species in Italy which encourages further sampling in the mountain regions across Europe (Alsammar *et al*. 2019). These findings indicate that these substrates may not be the natural niche of the *Saccharomyces* species, a theory that contradicts the adaptation model, which postulates that for an organism to be adapted to a niche, it must be abundant in that niche (Goddard and Greig 2015). Given the low abundance and habitat diversity of *S. cerevisiae*, it has been proposed that is it a nomad, that is not adapted to a specific niche. Although the nomad model was applied to *S. cerevisiae*, the criteria of this model, such as the presence of species in low abundance, could also be applied to the other species of the *Saccharomyces* genus (Goddard and Greig 2015). Extensive sampling of various habitats is needed to confirm the nomad nature of the wild *Saccharomyces* species.

## CONCLUSIONS AND PERSPECTIVES

Domestication processes have contributed greatly to the evolution of genome *Saccharomyces* species (Gallone *et al*. 2016; Dujon and Louis 2017). In the last few decades, researchers started to discover a large biodiversity of *Saccharomyces* species in the natural environment, prompting to focus their studies on the ecology and distribution of wild species (Sniegowski, Dombrowski and Fingerman 2002; Sampaio and Goncalves 2008; Charron *et al*. 2014b; Kowallik, Miller and Greig 2015; Sylvester *et al*. 2015; Kowallik and Greig 2016; Alsammar *et al*. 2019), genome evolution of the *Saccharomyces* species and their hybrids (Dunn and Sherlock 2008; Morales and Dujon 2012; Piatkowska *et al*. 2013; Hewitt *et al*. 2014; Dujon and Louis 2017; Peris *et al*. 2018), population genomics (Liti *et al*. 2009; Schacherer *et al*. 2009; Louis 2011; Peter *et al*. 2018) and phenotype variation (Warringer *et al*. 2011; Naseeb *et al*. 2016). The feasibility of whole-genome sequencing allowed the redefinition of the *Saccharomyces* species taxonomy based on the phylogeny rather than the concept of reproductive isolation and helped the identification of diverged populations of the yeast's species and strains according to their geography, environmental niche and human domestication (Peter *et al*. 2018).

Species belonging to the *Saccharomyces* genus are now known to be residing in soil, bark, decaying leaves, insect guts and in healthy and diseased human guts. The optimization of isolation techniques allowed the detection of new species and targeted metagenomic approaches were able to assess the degree of *Saccharomyces* species biodiversity present in the wild. For further insights on the natural history and evolution of *Saccharomyces* species more sampling of novel niches in different regions of the world would be desirable.
